# The cytoplasmic LSm1-7 and nuclear LSm2-8 complexes exert opposite effects on Hepatitis B virus biosynthesis and interferon responses

**DOI:** 10.3389/fimmu.2022.970130

**Published:** 2022-08-09

**Authors:** Naimur Rahman, Jiazeng Sun, Zhili Li, Aryamav Pattnaik, Rodrigo Mohallem, Mengbo Wang, Majid Kazemian, Uma K. Aryal, Ourania Andrisani

**Affiliations:** ^1^ Department of Basic Medical Sciences, Purdue University, West Lafayette, IN, United States; ^2^ Purdue Center for Cancer Research, Purdue University, West Lafayette, IN, United States; ^3^ Department of Biochemistry, Purdue University, West Lafayette, IN, United States; ^4^ Purdue Proteomics Facility, Bindley Bioscience Center, Purdue University, West Lafayette, IN, United States; ^5^ Department of Comparative Pathobiology, Purdue University, West Lafayette, IN, United States; ^6^ Department of Computer Science, Purdue University, West Lafayette, IN, United States

**Keywords:** Hepatitis B Virus (HBV), LSm1-7 and LSm2-8 complexes, *N*6-adenosine methylation (m^6^A), Cp028, Methylated RNA immunoprecipitation (MeRIP)

## Abstract

Despite many studies on host or viral gene expression, how the cellular proteome responds to internal or external cues during the infection process remains unclear. In this study, we used a Hepatitis B Virus (HBV) replication model and performed proteomic analyses to understand how HBV evades innate immunity as a function of cell cycle progression. Specifically, we performed proteomic analyses of HBV-replicating cells in G1/S and G2/M phases, as a function of IFN-α treatment. We identified that the conserved LSm (Like-Sm1-8) proteins were differentially regulated in HBV replicating cells treated with IFN-α. Specifically, in G2/M phase, IFN-α increased protein level of LSm1, the unique subunit of cytoplasmic LSm1-7 complex involved in mRNA decay. By contrast, IFN-α decreased LSm8, the unique subunit of nuclear LSm2-8 complex, a chaperone of U6 spliceosomal RNA, suggesting the cytoplasmic LSm1-7 complex is antiviral, whereas the nuclear LSm2-8 complex is pro-viral. In HBV replication and infection models, siRNA-mediated knockdown of LSm1 increased all viral RNAs. Conversely, LSm8 knockdown reduced viral RNA levels, dependent on *N*6-adenosine methylation (m^6^A) of the epsilon stem-loop at the 5′ end of pre-Core/pregenomic (preC/pg) RNA. Methylated RNA immunoprecipitation (MeRIP) assays demonstrated reduced viral RNA methylation by LSm8 knockdown, dependent on the 5’ m6A modification, suggesting the LSm2-8 complex has a role in mediating this modification. Interestingly, splicing inhibitor Cp028 acting upstream of the LSm2-8 complex suppressed viral RNA levels without reducing the 5’ m6A modification. This observation suggests Cp028 has novel antiviral effects, likely potentiating IFN-α-mediated suppression of HBV biosynthesis.

## Introduction

Chronic Hepatitis B virus (HBV) infection is associated with liver fibrosis, cirrhosis and development of hepatocellular carcinoma (HCC) (1). Despite the availability of an efficacious preventive vaccine, the World Health Organization reports more than 250 million people are chronically infected with HBV, having increased risk for developing HCC. Current treatments for HBV infection (e.g. nucleoside analogs or interferons) efficiently reduce viremia but are ineffective in persistently suppressing viral replication. Nucleoside analogs result in viral resistance, whereas pegylated Interferon-α (IFN-α) which stimulates innate immune responses against the virus, has less than 20% cure rate (2). New and effective therapies are needed to achieve viral clearance. Herein, we report a novel mechanism involved in the IFN-α effect on HBV biosynthesis.

HBV is a non-cytopathic, enveloped, hepadnavirus that contains a 3.2 kb partially double-stranded DNA genome, the relaxed circular DNA (rcDNA), and replicates *via* reverse transcription of the 3.5Kb pregenomic (pg) RNA. HBV uses sodium taurocholate co-transporting polypeptide (NTCP) receptor to enter hepatocytes (3, 4). After uncoating, HBV capsids transport viral rcDNA to the nucleus, and following DNA repair, rcDNA forms the covalently closed-circular DNA (cccDNA), template for all viral transcripts. Viral RNAs have different starting sites, but all terminate at a single/common polyA site. All viral RNAs contain the epsilon (ϵ) stem-loop at their 3’ end, while only the 3.5Kb pgRNA and preC-RNA contain the epsilon stem-loop at both ends. PreC-RNA encodes the pre-core/HBeAg protein. pgRNA serves as mRNA for translation of core antigen (HBc) and viral polymerase (P), as well as template for reverse transcription following encapsidation (5, 6). It remains to be determined what regulates the balance between pgRNA translation/degradation and encapsidation (7, 8). N6-adenosine methylation (m^6^A) of the epsilon stem-loop at the 5′ end of pgRNA is required for pgRNA encapsidation (9), whereas m^6^A of the epsilon sequence at the 3’ end of all viral RNAs is linked to IFN-α-mediated RNA degradation by the 3’ to 5’ ISG20 RNA exonuclease (10, 11).

The life cycle of HBV and its interaction with the host have been studied extensively, by *in vitro* replication and infection models (5, 12, 13), as well as animal models (14). Despite the wealth of studies on HBV biosynthesis, new technologies continue to generate further understanding of the infection process, and of the parameters that regulate HBV life cycle and disease pathogenesis. For example, RNAseq analyses of HBV infected primary human hepatocytes (PHHs) identified several pro-viral host factors upregulated by HBV infection in the G2/M phase (15). Likewise, a genome-wide gain-of-function screen employing a poorly permissive hepatoma cell line identified CDKN2C as a host factor for HBV replication, functioning in G1/S phase (16). The results of these two *in vitro* studies identified distinct molecules having a regulatory role in HBV biosynthesis, likely reflecting different physiological contexts during the infection process. However, despite various studies on host or viral gene expression, how the host cellular proteome responds to HBV replication or the underlying mechanism and functional significance of such responses during viral replication remain elusive.

In this study, we employed the *in vitro* HBV replication model of HepAD38 cells (12) and performed proteomic analyses of HBV replicating cells as a function of cell cycle progression and IFN-α treatment. This HBV replication model is suitable for proteomic analyses because all cells replicate the virus. In addition, synchronization of cells in culture in G1/S and G2/M phases of the cell cycle can provide specific information of how HBV infection in combination with IFN-α alters the hepatocyte proteome.

We report herein identification of a novel set of host proteins differentially regulated during HBV replication and by IFN-α. These proteins are members of the highly conserved LSm (like Sm) family that forms circular, RNA-binding hetero-heptameric complexes (17). The cytoplasmic LSm1-7 complex initiates mRNA decay. The nuclear LSm2-8 complex acts as chaperone for U6 spliceosomal RNA (17). Interestingly, the LSm proteins were initially identified in serum from patients with autoimmune systemic lupus erythematosus (18, 19), suggesting a link of their expression to inflammation. However, their involvement in the IFN-α response to HBV infection has been unknown. Herein, we report the novel observation of the involvement of both cytoplasmic LSm1-7 and nuclear LSm2-8 complex in modulating HBV biosynthesis.

## Materials and methods

### Cell culture, transfections and HBV infection

HepAD38 cells grown in the presence of tetracycline, as described (12). Cells routinely tested for mycoplasma. Authentication of the HepAD38 cell line by short tandem repeat (STR) analysis performed by ATCC. HepG2 cells maintained in DMEM/F12 supplemented with 10% FBS, transfected with whole HBV genome 1.3mer plasmids, pHBV-WT, pHBV-M1, pHBV-M2 and pHBV-M3, kindly provided by Dr. A. Siddiqui (20). MeRIP assays performed as described (20). Virus preparation from HepAD38 cells (21) and HBV infection of HepG2-NTCP cells carried out as described (22), except 4% polyethelene glycol (PEG) was included in infected cell media only during day1 of infection. Detailed protocols for virus preparation and infection included in Supplementary Materials section.

### Cell synchronization and treatments

Cell Synchronization of HepAD38 cells performed by the double thymidine block as described (23). Detailed protocol included in Supplementary Materials section. Whole cell extracts (WCE) prepared from G1/S and G2/M synchronized cultures, and processed for liquid chromatography (LC)-mass spectrometry (MS and MS/MS) as described (24). Three independent WCE preparations similarly prepared and analyzed by LC-MS/MS.

Nuclear extract preparation, Size Exclusion Chromatography (SEC), Proteomics Sample Preparation, Liquid Chromatography-Tandem Mass Spectrometry (LC-MS/MS) analysis and Mass Spectrometry Data Analysis described in detailed protocols included in Supplementary Materials section.

Immunoblots, siRNA transfections and RT-PCR quantification performed as previously described (25, 26). **Supplementary Tables S1–S4** list plasmids, siRNA sequences, antibodies, primer sequences for qRT-PCR quantification, reagents, chemicals and kits.

### Flow cytometry

HBV infected HepG2-NTCP cells on day 7 post-infection analyzed by flow cytometry in an Attune NxT Flow Cytometer (Thermo Fisher) using HBc and HBsAg antibodies. Data analysis performed using FlowJo software 10.8.1. Detailed flow cytometry protocol included in Supplementary Materials section.

### Statistical analysis

Statistical analysis performed using unpaired *t* test in GraphPad Prism version 6.0 (GraphPad Software, San Diego, CA). Differences were considered significant when *p* < 0.05.

## Results

### Proteomic alterations in HBV replicating hepatocytes during cell cycle progression and IFN-α treatment

**Figure 1:**
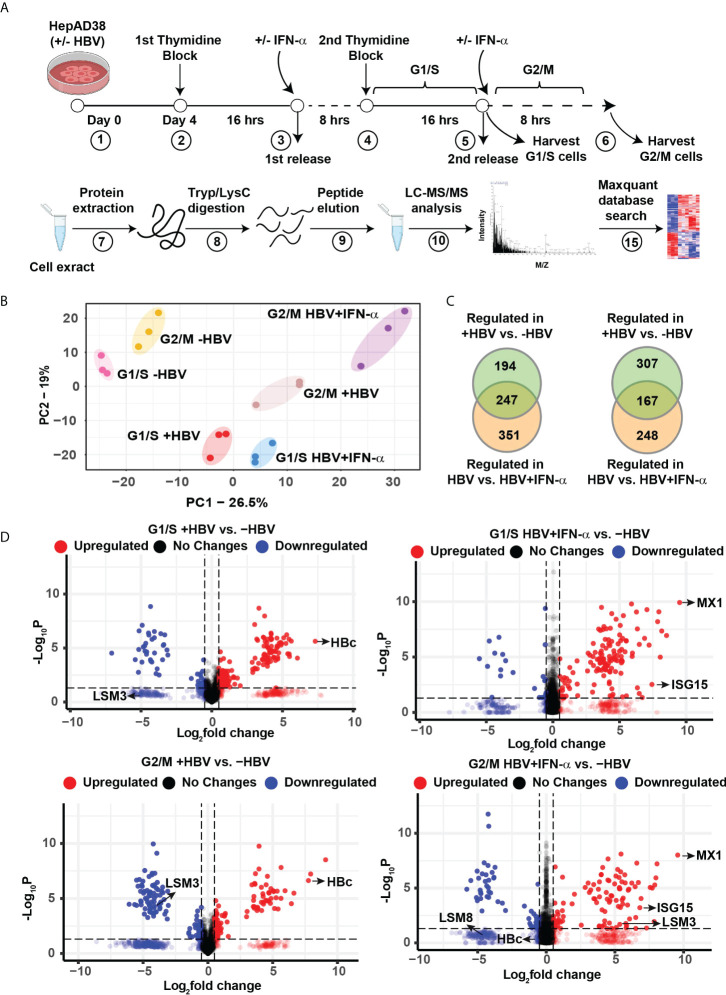
Label−free quantitative proteomic analysis of HBV replicating hepatocytes as a function of cell cycle progression and IFN-α treatment. **(A)** Experimental design and proteomics workflow. **(B)** Principal-component analysis (PCA) segregates the six experimental groups (n=3). **(C)** Ven diagram of differentially regulated protein (FC>2, p <0.05) in G1/S and G2/M as indicated, comparing HBV replicating vs. non-replicating cells and HBV replicating with (+) or without (-) INF-α (500 ng/ml) treatment. **(D)** Volcano plots showing distribution of differentially abundant proteins (FC>2, p <0.05) in G1/S and G2/M as indicated, comparing HBV replicating vs. non-replicating cells and HBV replicating with (+) or without (-) INF-α (500 ng/ml) treatment. Each dot represents a protein.

To investigate changes in protein composition of hepatocytes during HBV replication, we used the HepAD38 cell line that contains an integrated copy of the viral genome under control of tetracycline promoter (12). Following HBV replication for 4 days by tetracycline removal, cells were synchronized in G1/S and G2/M phases (23, 27). Treatment with IFN-α was for 24 h for G1/S synchronization, and an additional 8 h for synchronization in G2/M (**Figure 1A**). Following the workflow of **Figure 1A**, proteomics identified nearly 38,863 peptides mapped to 4575 proteins. From all proteins identified, 2858 proteins were quantified in at least 2 biological replicates from the same treatment group (**Supplementary dataset 1**). Principal-component analysis (PCA) of these quantified proteins showed tight clustering of treatment replicates and clear separation among all six experimental groups (**Figure 1B**), demonstrating high reproducibility of the global data. Our data also identified G1/S and G2/M checkpoint proteins upregulated in their respective cell cycle phases, thereby ensuring effective cell cycle synchronization (**Supplementary Figure S1**).

Next, we compared the proteome of 4-day HBV replicating *vs*. non-replicating cells, as a function of cell cycle progression, and IFN-α. In G1/S, the abundance of 441 proteins was significantly altered when comparing HBV replicating *vs.* non-replicating cells, and 598 proteins when HBV replicating cells were treated with IFN-α, of which, 247 proteins were common (FC>2, p <0.05) (**Figure 1C** and **Supplementary dataset 2**). In G2/M synchronized cells +/- HBV replication and IFN-α, the abundance of 474 proteins was significantly changed between HBV replicating *vs.* non-replicating cells, and 415 proteins when HBV replicating cells were treated with IFN-α, of which 167 proteins were common (FC>2, p <0.05) (**Figure 1C** and **Supplementary dataset 2**). The distribution of differentially regulated proteins, exhibiting significant variation in protein level (p < 0.05), is shown by the volcano plots (**Figure 1D**). In HBV replicating cells, one of the most upregulated proteins is the HBV core antigen (HBc), confirming replication of the virus (**Figure 1D**). As expected, IFN-α regulates expression of interferon responsive proteins (IFITM1, MX1, ISG15) (28) and several LSm proteins (17) (**Figure 1D**).

### Proteome of HBV replicating hepatocytes affected by IFN-α

**Figure 2:**
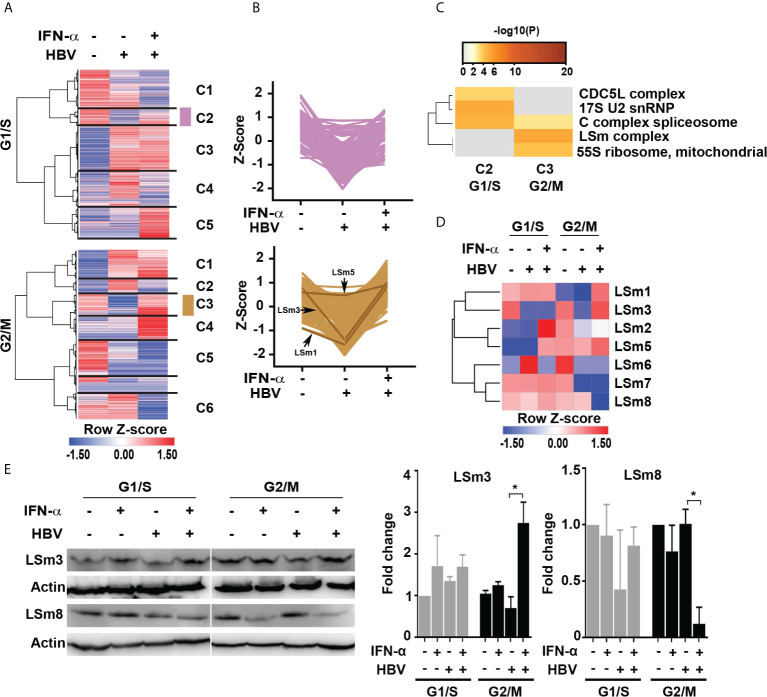
IFN-α regulated proteins in HBV replicating hepatocytes as a function of cell cycle progression. **(A)** Hierarchical clustering of significantly changed proteins as a function of HBV replication and IFN-α treatment in hepatocytes synchronized in G1/S and G2/M phases analyzed by one way ANOVA (p <0.05). **(B)** The changes in protein abundance (relative intensity value) of cluster C2 (112 proteins) of G1/S and C3 (157 proteins) of G2/M were normalized to Z score, using the Perseus platform (29). In G2/M C3, protein intensity of the LSm1, 3 and 5 subunits is shown. **(C)** Comparison of protein complex enrichment analyses of G1/S cluster C2 proteins and G2/M cluster C3 by the CORUM database. **(D)** Heatmap representation of LSm1-8 expression as a function of HBV replication and IFN-α treatment in hepatocytes synchronized in G1/S and G2/M phases. Color scale represents label free quantification (LFQ) Z-scored values. **(E)** Immunoblots of LSm3 and LSm8, using cell lysates from G1/S and G2/M synchronized HepAD38 cells, +/- HBV replication for 4 days and IFN-α (500 ng/ml) treatment, as indicated. A representative immunoblot is shown; (Right panels) quantification of LSm3 and LSm8 vs. actin, using unpaired t-test. *p<0.05.

To gain more insight into the pathways regulated by IFN-α in the context of HBV replication, we performed hierarchical clustering for identifying groups or clusters of proteins regulated similarly. We analyzed the total quantified proteins (4575 proteins) to determine statistically significant changes among the six treatment groups by one way ANOVA (p ≤ 0.05), as shown by the heatmaps (**Figure 2A** and **Supplementary dataset 3**). We found 1,192 proteins differentially regulated among all treatment groups. We observed five clusters in G1/S and six clusters in G2/M proteins. We focused on clusters C2 (112 proteins) of G1/S and C3 (157 proteins) of G2/M because they were comprised of proteins downregulated in the presence of HBV replication, and normalized or even upregulated upon addition of IFN-α, suggesting these proteins have anti-viral role (**Figure 2A**). The distribution of protein abundance across samples in clusters C2 of G1/S and C3 of G2/M is shown in **Figure 2B**.

Next, we employed the CORUM database (30, 31) that encompasses more than 4,000 experimentally characterized mammalian protein complexes. Identification of protein complexes provides the basis for understanding mechanisms of normal and diseased states, for example, how innate immunity counteracts viral infection. We interrogated clusters C2 and C3 from G1/S and G2/M, respectively. Based on the CORUM analysis, the G1/S C2 proteins were comprised of predicted components of catalytically active splicing complex (32), and the G2/M C3 proteins contained the LSm complex (**Figure 2C**).

We focused our investigation on the LSm complex because earlier studies demonstrated LSm proteins bind viral positive (+)-strand RNA genomes, promoting their translation and subsequently their replication (33, 34). Similar to (+)-strand RNA viruses (35), HBV replicates its genome *via* a RNA intermediate, the 3.5 Kb pgRNA that has dual functions, namely in mRNA translation and viral replication. Thus, we reasoned the LSm complexes may have a role in HBV biosynthesis. We compared LSm1 through LSm8 protein levels, forming the cytoplasmic LSm1-7 and nuclear LSm2-8 complexes (17), in HBV replicating cells *vs.* those treated with IFN-α (**Figure 2D**). The most robust changes in LSm protein levels were observed in G2/M, comparing HBV replicating cells in combination with IFN-α. Specifically, IFN-α increased protein levels of LSm1, 3 and 5 subunits (**Figure 2D**). By contrast, the most intriguing change was in protein level of LSm8, exhibiting robust and selective reduction upon IFN-α treatment in G2/M (**Figure 2D**). These proteomic results were validated by immunoblots, demonstrating that IFN-α increased protein level of LSm3 and reduced protein level of LSm8 in G2/M (**Figure 2E**).

### LSm complexes are involved in HBV biosynthesis

**Figure 3:**
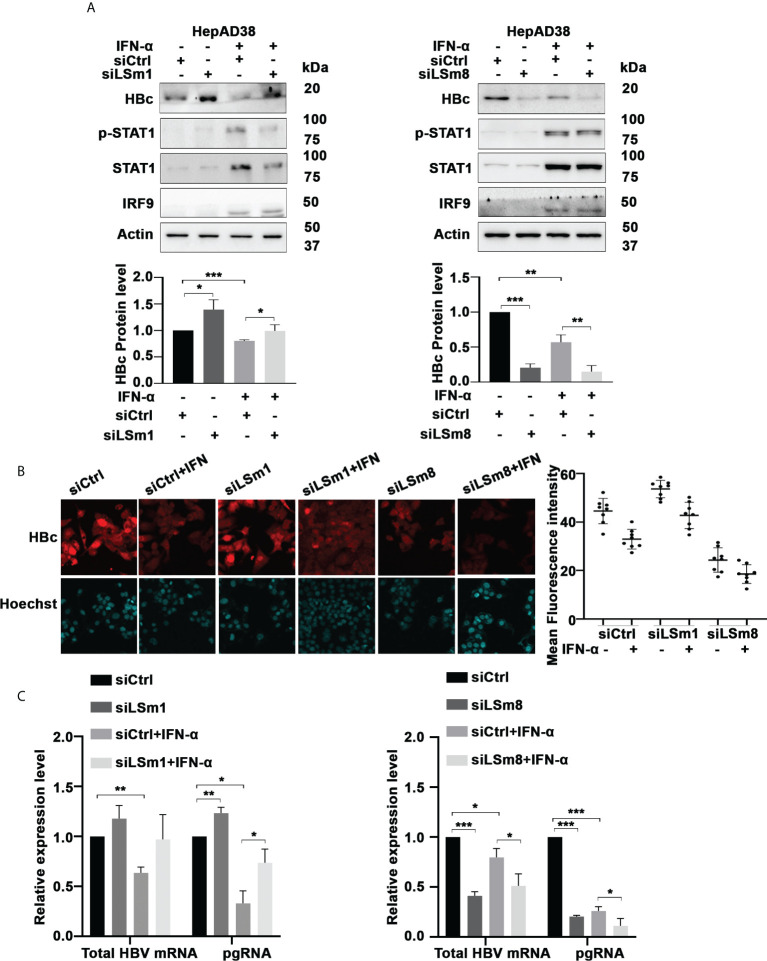
Role of Lsm complexes in HBV biosynthesis and IFN-α response. **(A)** Immunoblot of indicated proteins using lysates of unsynchronized HepAD38 cells replicating HBV for 4 days by tetracycline removal, treated with (+) or without **(-)** IFN-a (400ng/ml) for 4 days. Transfection of 50pM each of control siRNA (siCtrl), LSm1 siRNA (siLSm1) or LSm8 (siLSm8) at onset of HBV replication (n=4). (Panels below) Quantification of immunoblots by ImageJ software. n=4, *p<0.05, ** p<0.01, ***p<0.001. **(B)** HBc immunofluorescene microscopy of unsynchronized HepAD38 cells grown as in **(A)** (Right Panel) Quantification of HBc immunofluorescence by Image J software. **(C)** qRT-PCR of total HBV RNA and preC/pgRNA under conditions described in **(A)**. n=3, *p<0.05, ** p<0.01, ***p<0.001.

To determine the functional significance of the cytoplasmic and nuclear LSm complexes in HBV replication and antiviral IFN-α effect, we examined the effect of downregulation of LSm1 and LSm8 subunits on HBV biosynthesis. LSm1 and LSm8 subunits are unique subunits of the cytoplasmic LSm1-7 and nuclear LSm2-8 complexes respectively. Using HepAD38 cells, we determined protein level of HBc by immunoblots (**Figure 3A**) and immunofluorescence microscopy (**Figure 3B** and **Supplementary Figure S2A**), following knockdown of LSm1 and LSm8 by siRNA transfection and as a function of IFN-α treatment. Under the same conditions, we quantified HBV preC/pgRNA and total HBV RNA by qRT-PCR, using specific primers distinguishing preC/pgRNA from other RNAs (36) (**Figure 3C**). Interestingly, siLSm1 enhanced HBc protein and viral RNA levels, while it dampened the IFN-α effect. By contrast, siLSm8 significantly reduced HBc protein and viral RNA levels, and potentiated the anti-viral IFN-α effect (**Figures 3A, C**). Activation of IFN-α signaling was determined by immunoblots of pStat1/Stat1 and IRF9 induction (**Figure 3A**) and induction of ISGs (**Supplementary Figure S2B**).

**Figure 4:**
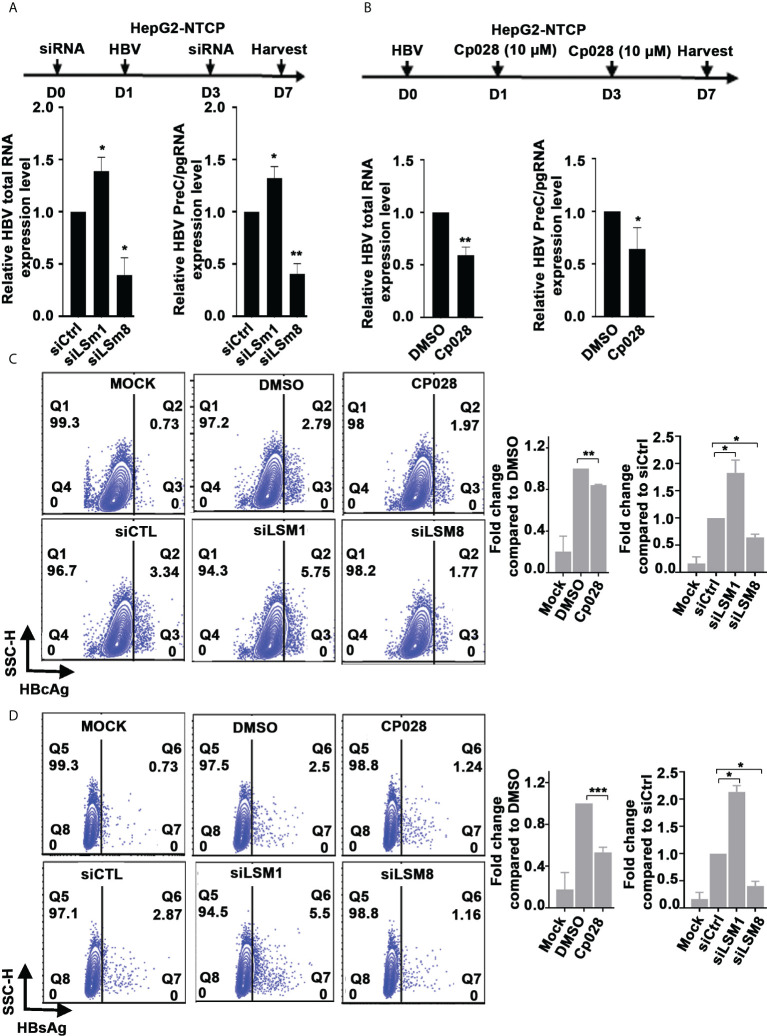
Role of LSm complexes in HBV biosynthesis using an HBV infection model. **(A)** Diagram shows treatment timeline of HepG2-NTCP cells. On day 0, cells were transfected with 50pM of indicated siRNAs (siCtrl, siLSm1, or siLSm8), infected with 100vge/cell of HBV on day1, followed by another siRNA transfection on day 3. Cells harvested on day7 for preparation of RNA and qRT-PCR analyses of total HBV RNA and preC/pg RNA. n=3, *p<0.05, ** p<0.01 **(B)** Timeline of HepG2-NTCP infection with HBV (100vge/cell) and treatment with Cp028 (10µM). Cells harvested on day7 for preparation of RNA and qRT-PCR analyses of total HBV RNA and preC/pg RNA. n=3, *p<0.05, ** p<0.01  **(C, D).** Flow cytometric quantification of HBV infected HepG2-NTCP cells (500vge/cell), on day7 post-infection under conditions described in **(A)**, using anti-HBc **(C)** or anti-HBsAg **(D)** Mock, indicates uninfected cells; DMSO added to HBV infected cells as vehicle control for Cp028 addition to HBV infected cells. A representative image shown. Right panels are quantification of HBc-positive and HBsAg-positive cells from three independent experiments. *p<0.05, **p<0.01, ***p<0.001.

Similarly, in the context of HBV infection of HepG2-NTCP cells, siLSm1 increased levels of all viral RNAs, whereas siLSm8 had the opposite effect (**Figure 4A**). The LSm2-8 complex in association with small nuclear RNAs U4/U6 and U5, and small nuclear proteins forms the pre-catalytic spliceosomal B complex (37). The splicing inhibitor Cp028 suppresses conversion of pre-catalytic spliceosomal B complex into the activated B^act^ complex (38). We reasoned, use of Cp028, in the context of HBV replication would reveal aspects of the mechanism by which the LSm2-8 complex regulates HBV replication. In HBV infected HepG2-NTCP cells, Cp028 reduced expression of all viral RNAs including preC/pgRNAs (**Figure 4B**). We further confirmed these results by flow cytometric quantification of HBc and HBsAg on day 7 post-infection (**Figures 4C, D** and **Supplementary Figure S3**). Cp028 and siLSm8 reduced the number of HBV infected cells expressing HBc (**Figure 4C**) or HBsAg (**Figure 4D**), while siLSm1 exerted the opposite effect (**Figures 4C, D**). Importantly, neither siLSm1 and siLSm8, nor Cp028 had an effect on cell viability (**Supplementary Figure S2C**).

### Opposite effects of the LSm1-7 vs. LSm2-8 complexes on HBV biosynthesis

**Figure 5:**
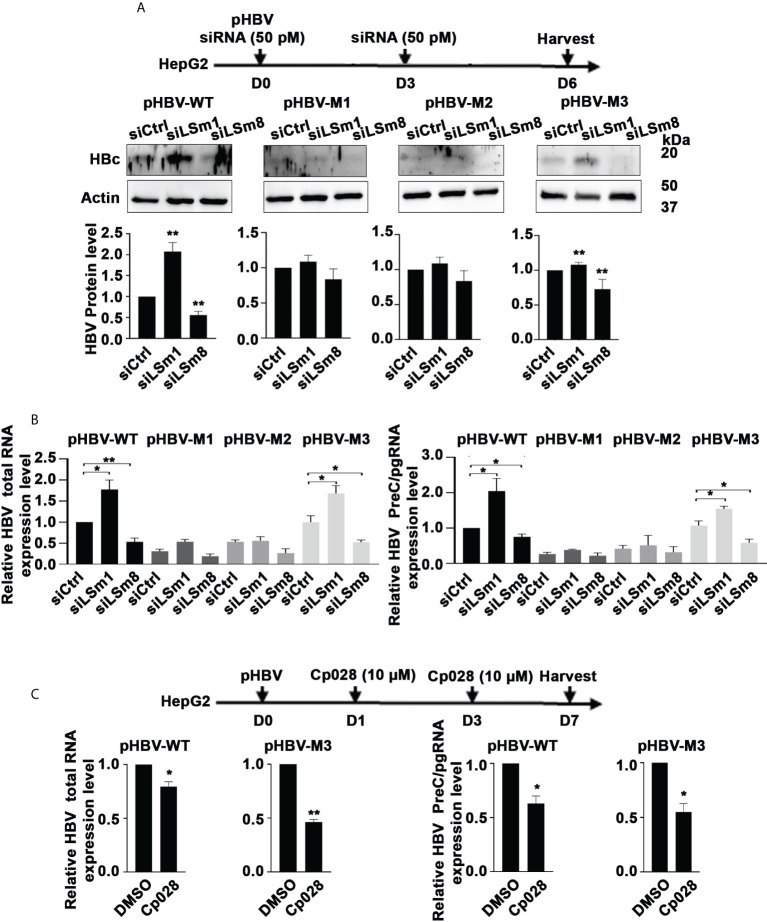
Opposite effects of the LSm1-7 *vs.* LSm2-8 complexes on HBV biosynthesis. **(A)** Timeline of transfections of 1.3mer HBV plasmid DNA and 50 pM of indicated siRNAs in HepG2 cells. pHBV-WT, pHBV-M1, pHBV-M2 and pHBV-M3 (1.0µg each per 6-well plate) transfected by lipofectamine3000 in HepG2 cells. HBc immunoblot of day6-transfected HepG2 lysates. Panels below immunoblots show quantification of HBc by ImageJ software from three independent experiments. *p<0.05, **p<0.01 **(B)** RNA isolated from transfected cells in **(A)** used for RT-PCR quantification of total HBV RNA and preC/pgRNA. n=3 *p<0.05, **p<0.01 C. Timeline of transfection of 1.3mer HBV plasmid DNA in HepG2 cells, in combination with Cp028 (10µM) treatment. pHBV-WT and pHBV-M3 (1.0µg each per 6-well plate) transfected by lipofectamine3000 in HepG2 cells. HBc immunoblot of HepG2 lysates shown in **Supplementary Figure S5**. RNA isolated from transfected cells in **(C)** used for RT-PCR quantification of total HBV RNA and preC/pgRNA. n=3 *p<0.05, **p<0.01.

The proteomic results of **Figure 2** suggest that IFN-α interferes with the LSm complexes. Since aspects of the antiviral IFN-α effect require the m6A modification for ISG20-mediated degradation of the viral transcripts (10, 20), we tested whether LSm1-7 and LSm2-8 complexes regulate m6A modifications of the epsilon structure of HBV RNAs. We transfected plasmids containing the 1.3mer of the WT HBV genome or the HBV mutants M1 (5’ and 3’ m6A mutations), M2 (5’ mutation) and M3 (3’mutation) generated by the Siddiqui lab (20) in combination with siLSm1 or siLSm8. We quantified HBc protein levels by immunoblots (**Figure 5A**) and immunofluorescence microscopy (**Supplementary Figure S4**), and total HBV RNA and preC/pgRNA by qRT-PCR (**Figure 5B**). In pHBV-WT and pHBV-M3 transfected cells, siLSm1 enhanced HBc and viral RNA levels, whereas siLSm8 exerted the opposite effect, i.e., siLSm8 decreased HBc (**Figure 5A**) and viral RNAs (**Figure 5B**), suggesting the Lsm2-8 complex may have a role on the 5’m6A modification. By contrast M1 and M2 HBV genomes lacking the 5’ m6A modification, do not exhibit a significant effect by either siLSm1 or siLSm8 (**Figures 5A, B**). Since siLSm1 enhanced steady state level of all HBV RNAs (**Figure 5B**), the cytoplasmic LSm1-7 complex likely regulates HBV RNA decay, modulating mRNA translation *vs*. mRNA decay (39). Cp028 acting upstream of the LSm2-8 complex reduced HBc protein (**Supplementary Figure S5**) and RNA levels only from pHBV-WT and pHBV-M3 genomes (**Figure 5C**).

### LSm2-8 complex is required for the 5’ m6A modification

**Figure 6:**
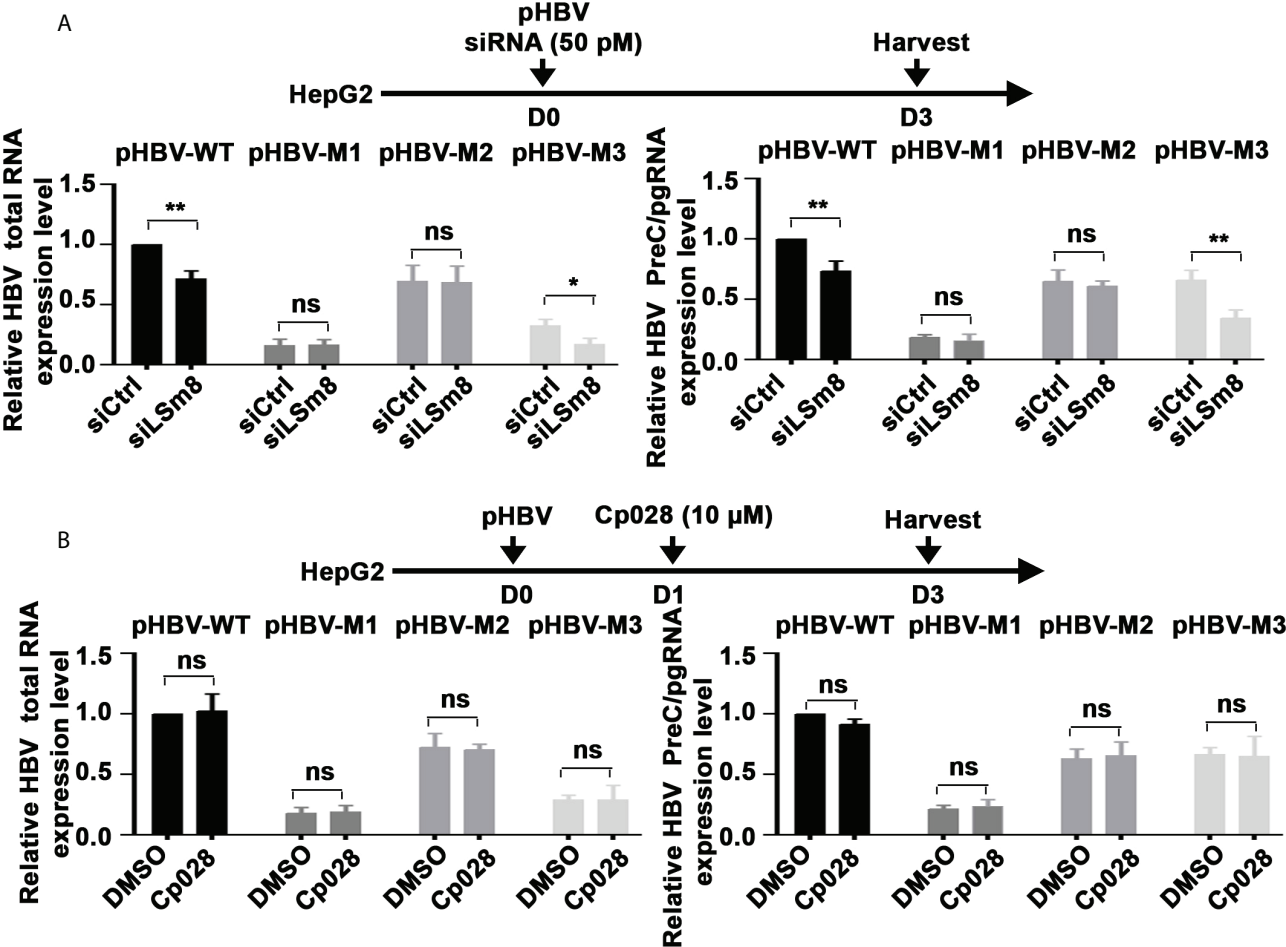
The LSm2-8 complex is required for the 5’ m6A modification. **(A)** and **(B)** Timeline of transfections of 1.3mer HBV plasmid DNA and 50 pM of indicated siRNAs in HepG2 cells **(A)** or with addition of 10µM Cp028 **(B)**. pHBV-WT, pHBV-M1, pHBV-M2 and pHBV-M3 (1.0µg each per 6-well plate) transfected by lipofectamine3000 in HepG2 cells. RNA extracted from day3 transfected HepG2 cells, immunoprecipitated with anti-m6A or IgG, and total HBV RNA and preC/pgRNA quantified by RT-PCR. Results expressed relative to IgG. n=3 *p<0.05, **p<0.01. ns, not significant.

To determine whether the LSm2-8 complex has a role in the 5’ m6A modification of the preC/pgRNA, we carried out methylated RNA immunoprecipitation (MeRIP) assays. Plasmids encoding the WT HBV genome or HBV mutants M1, M2 and M3 (20) were transfected in HepG2 cells, in combination with siLSm8 transfection, or Cp028 addition. MeRIP assays were performed with m6A-specific antibody or IgG, and immunoprecipitated RNA quantified by qRT-PCR. Viral RNAs expressed from pHBV-WT and pHBV-M3 genomes exhibited reduced m6A modification upon LSm8 knockdown (**Figure 6A**). Interestingly, siLSm8 reduced the level of m6A-modified RNA expressed from pHBV-M3 to a level similar with the methylation deficient pHBV-M1, lacking both 5’ and 3’ mA6 modifications. We interpret these results to mean the LSm2-8 complex is involved in mediating the 5’ epsilon m6A modification required for pgRNA encapsidation (9). By contrast, Cp028 had no effect on the m6A modifications of the viral RNAs (**Figure 6B**), indicating that Cp028 reduces viral RNA levels (**Figure 4B**) by a mechanism not involving the 5’ m6A modification.

**Figure 7:**
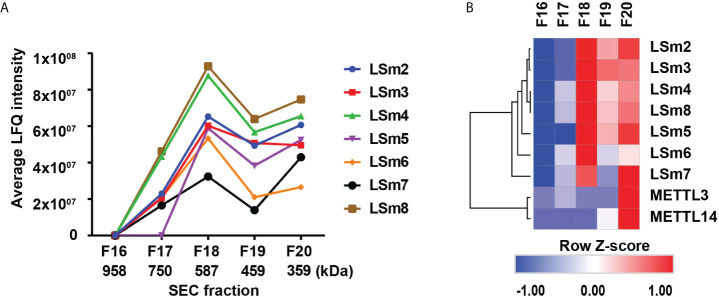
Detection of nuclear LSm2-8 complex in HepaRG lysates. **(A)** Native nuclear extracts from HepaRG cells fractionated by Superdex 30 size exclusion chromatography (SEC). Fractions # 17-20 analysed by LC MS/MS. LSm2-8 complex proteins eluted in fractions #17-20, peaking in fraction 18, with an estimated native molecular mass of 587 kDa. **(B)** Hierarchical clustering analyses of select proteins from SEC fractions # 17-20. Color scale represents Z-scored LFQ values.

In further support of these observations, we analyzed native nuclear extracts from HepaRG cells (40) by size exclusion chromatography, followed by label-free quantitative mass spectrometry analysis of the proteins eluting in fractions 16-20 (**Figure 7A**). The heatmap of duplicate samples of proteins identified by mass spectrometry, and the Pearson correlation co-efficient analyses of duplicate samples demonstrate the quality of the analysis (**Supplementary Figure S6**). LSm2-8 proteins eluted in fractions #17-20 (**Figure 7A**), with an estimated native molecular mass of 587 kDa for fraction #18 to 359 kDa for fraction #20, suggesting the LSm2-8 hetero-heptameric complex associates with various other proteins. Interestingly, the heatmap of specific proteins identified by mass spectrometry in fractions #17-20 showed that fraction #20 contains all the subunits of the LSm2-8 complex and co-elutes with various other proteins (not shown) including the RNA methyltransferases METTL3/14 (**Figure 7B**). These results suggest that the LSm2-8 complex likely serves as scaffold for the epi-transcriptomic modifications of the preC/pgRNA.

## Discussion

Herein, we described a proteomics study using HBV replicating cells, synchronized in G1/S or G2/M phases of the cell cycle, and treated with IFN-α. Our analyses identified nearly 38,863 peptides mapped to 4,575 proteins (**Supplementary dataset 1**); employing established bioinformatic approaches, we identified predicted protein complexes that exhibited statistically significant differences, upregulated or downregulated, in our comparison groups. We identified 1192 differentially regulated proteins (p ≤ 0.05) displaying statistically significant changes in G1/S and G2/M phases, as a function of HBV replication and IFN-α (**Figure 2**). Since proteins within each cluster have a mechanistic link, we identified by CORUM analyses predicted protein complexes (**Figure 2C**) exhibiting changes in their protein level during HBV replication and IFN-α treatment. These CORUM analyses revealed that in G1/S phase significant complexes are comprised of proteins involved in the catalytically active splicing C complex (32). We speculate that by this mechanism, HBV likely suppresses splicing events involving pgRNA, which would be detrimental to the virus, reversed by IFN-α. Significantly, HBV infection alters or suppresses cellular splicing as an additional mechanism contributing to oncogenic transformation (41). Further studies are required to decipher this mechanism.

In G2/M phase, the most significant complex identified by the CORUM analyses was the LSm complex (**Figure 2C**). Cellular LSm complexes include the cytoplasmic Lsm1–7 complex involved in the 5’ to 3’ mRNA decay (17), and the nuclear LSm2-8 complex acting as chaperone for U6 spliceosomal RNA (17). In G2/M, LSm1 protein levels were downregulated by HBV replication and upregulated by IFN-α, while IFN-α dramatically reduced LSm8 protein level (**Figures 2D, E**). Based on these observations, we proceeded to knockdown the unique subunits of the cytoplasmic and nuclear complexes, LSm1 and LSm8 proteins, respectively. LSm1 knockdown enhanced HBV biosynthesis and dampened the antiviral IFN-α effect. By contrast, LSm8 knockdown repressed viral biosynthesis and further promoted the antiviral IFN-α effect (**Figure 3**). We interpret these results to mean disruption of the cytoplasmic LSm1-7 mRNA decay complex alleviates viral RNA degradation. Our observation agrees with earlier studies demonstrating that deletion of LSm1 results in accumulation of deadenylated mRNAs with intact cap structure at their 5’-terminus (42).

Regarding the role of the nuclear LSm2-8 complex in HBV biosynthesis, our results show: 1) knockdown of LSm8 reduces the 5’m6A modification, based on MeRIP assays using the pHBV-M3 genome (**Figure 6A**); and 2) the LSm2-8 complex co-elutes with the RNA methyltransferases METTL3/14 (**Figure 7B**) that mediate the 5’ m6A modification required for pgRNA encapsidation (9). We speculate the RNA binding LSm2-8 complex has a role in the recognition and protection of the epsilon structure of the preC/pgRNA, enabling the 5’m6A modification. We base this hypothesis on recent studies demonstrating involvement of the LSm2-8 complex in the recognition and protection of correctly folded TER1 non-coding RNA to ensure telomerase assembly and activity (43). Alternatively, the LSm2-8 complex may serve as scaffold for recruitment of METTL3/14. 3) Another interesting observation from our studies is the distinct effect of the splicing inhibitor Cp028 (38) in reducing viral RNA levels (**Figure 4**) without interfering with the m6A modifications of viral RNAs (**Figure 6B**). We interpret these results to suggest that combined treatment with IFN-α and Cp028 could potentiate the antiviral IFN-α effect. Additional studies are required to understand the precise role of these complexes vis-à-vis the Cp028 effect in the HBV life cycle and IFN-α response.

## Data availability statement

The datasets presented in this study can be found in online repositories. The names of the repository/repositories and accession number(s) can be found below: MassIVE data repository (massive.ucsd.edu/), with ID: MSV000089600.

## Author contributions

NR performed proteomics. JS and ZL infection studies. AP, MW and MK flow cytometry. RM and UA proteomics. OA supported research and wrote manuscript. All authors contributed to the article and approved the submitted version.

## Funding

This work supported by NIH grants R35GM138283 to MK and DK044533 to OA. Supported by NIH grant P30CA023168 to Purdue Center for Cancer Research, and NIH/NCRR RR025761.

## Conflict of interest

The authors declare that the research was conducted in the absence of any commercial or financial relationships that could be construed as a potential conflict of interest.

## Publisher’s note

All claims expressed in this article are solely those of the authors and do not necessarily represent those of their affiliated organizations, or those of the publisher, the editors and the reviewers. Any product that may be evaluated in this article, or claim that may be made by its manufacturer, is not guaranteed or endorsed by the publisher.

## References

[1] El-Serag HB. Epidemiology of viral hepatitis and hepatocellular carcinoma. Gastroenterology (2012) 142:1264–1273.e1261. doi: 10.1053/j.gastro.2011.12.061 22537432 PMC3338949

[2] Pierra Rouviere C Dousson CB Tavis JE. HBV replication inhibitors. Antiviral Res (2020) 179:104815. doi: 10.1016/j.antiviral.2020.104815 32380149 PMC7293572

[3] Ni Y Lempp FA Mehrle S Nkongolo S Kaufman C Falth M. hepatitis B and D viruses exploit sodium taurocholate co-transporting polypeptide for species-specific entry into hepatocytes. Gastroenterology (2014) 146:1070–83. doi: 10.1053/j.gastro.2013.12.024 24361467

[4] Yan H Zhong G Xu G He W Jing Z Gao Z. Sodium taurocholate cotransporting polypeptide is a functional receptor for human hepatitis B and d virus. Elife (2012) 1:e00049. doi: 10.7554/eLife.00049 23150796 PMC3485615

[5] Seeger C Mason WS. Molecular biology of hepatitis B virus infection. Virology (2015) 479-480C:672–86. doi: 10.1016/j.virol.2015.02.031 PMC4424072 25759099

[6] Hu J Protzer U Siddiqui A. Revisiting hepatitis B virus: Challenges of curative therapies. J Virol (2019) 93(20):e01032–19. doi: 10.1128/JVI.01032-19 31375584 PMC6798116

[7] Ryu D-K Kim S Ryu W-S. hepatitis B virus polymerase suppresses translation of pregenomic RNA *via* a mechanism involving its interaction with 5′ stem–loop structure. Virology (2008) 373:112–23. doi: 10.1016/j.virol.2007.11.010 18155120

[8] Yao Y Yang B Cao H Zhao K Yuan Y Chen Y. RBM24 stabilizes hepatitis B virus pregenomic RNA but inhibits core protein translation by targeting the terminal redundancy sequence. Emerg Microbes Infect (2018) 7:86. doi: 10.1038/s41426-018-0091-4 29760415 PMC5951808

[9] Kim GW Moon JS Siddiqui A. N6-methyladenosine modification of the 5' epsilon structure of the HBV pregenome RNA regulates its encapsidation by the viral core protein. Proc Natl Acad Sci USA (2022) 119(7):e2120485119. doi: 10.1073/pnas.2120485119 35135882 PMC8851549

[10] Liu Y Nie H Mao R Mitra B Cai D Yan R. Interferon-inducible ribonuclease ISG20 inhibits hepatitis B virus replication through directly binding to the epsilon stem-loop structure of viral RNA. PloS Pathog (2017) 13:e1006296. doi: 10.1371/journal.ppat.1006296 28399146 PMC5388505

[11] Imam H Kim GW Mir SA Khan M Siddiqui A. Interferon-stimulated gene 20 (ISG20) selectively degrades N6-methyladenosine modified hepatitis B virus transcripts. PloS Pathog (2020) 16:e1008338. doi: 10.1371/journal.ppat.1008338 32059034 PMC7046284

[12] Ladner SK Otto MJ Barker CS Zaifert K Wang GH Guo JT. Inducible expression of human hepatitis B virus (HBV) in stably transfected hepatoblastoma cells: a novel system for screening potential inhibitors of HBV replication. Antimicrob Agents Chemother (1997) 41:1715–20. doi: 10.1128/AAC.41.8.1715 PMC163991 9257747

[13] Lucifora J Durantel D Belloni L Barraud L Villet S Vincent IE. Initiation of hepatitis B virus genome replication and production of infectious virus following delivery in HepG2 cells by novel recombinant baculovirus vector. J Gen Virol (2008) 89:1819–28. doi: 10.1099/vir.0.83659-0 18632952

[14] Burwitz BJ Zhou Z Li W. Animal models for the study of human hepatitis B and d virus infection: New insights and progress. Antiviral Res (2020) 182:104898. doi: 10.1016/j.antiviral.2020.104898 32758525

[15] Xia Y Cheng X Li Y Valdez K Chen W Liang TJ. hepatitis B virus deregulates the cell cycle to promote viral replication and a premalignant phenotype. J Virol (2018) 92(19):e00722–18. doi: 10.1128/JVI.00722-18 30021897 PMC6146796

[16] Eller C Heydmann L Colpitts CC El Saghire H Piccioni F Jühling F. A genome-wide gain-of-function screen identifies CDKN2C as a HBV host factor. Nat Commun (2020) 11:2707. doi: 10.1038/s41467-020-16517-w 32483149 PMC7264273

[17] Montemayor EJ Virta JM Hayes SM Nomura Y Brow DA Butcher SE. Molecular basis for the distinct cellular functions of the Lsm1-7 and Lsm2-8 complexes. Rna (2020) 26:1400–13. doi: 10.1261/rna.075879.120 PMC7491322 32518066

[18] Tan EM Kunkel HG. Characteristics of a soluble nuclear antigen precipitating with sera of patients with systemic lupus erythematosus 1966. J Immunol (2006) 96:464–71. 5932578

[19] Tsokos GC. In the beginning was Sm. J Immunol (2006) 176:1295–6. doi: 10.4049/jimmunol.176.3.1295 16424152

[20] Imam H Khan M Gokhale NS McIntyre ABR Kim GW Jang JY. N6-methyladenosine modification of hepatitis B virus RNA differentially regulates the viral life cycle. Proc Natl Acad Sci USA (2018) 115:8829–34. doi: 10.1073/pnas.1808319115 PMC6126736 30104368

[21] Watashi K Liang G Iwamoto M Marusawa H Uchida N Daito T. Interleukin-1 and tumor necrosis factor-α trigger restriction of hepatitis B virus infection *via* a cytidine deaminase activation-induced cytidine deaminase (AID). J Biol Chem (2013) 288:31715–27. doi: 10.1074/jbc.M113.501122 PMC3814766 24025329

[22] Michailidis E Pabon J Xiang K Park P Ramanan V Hoffmann HH. A robust cell culture system supporting the complete life cycle of hepatitis B virus. Sci Rep (2017) 7:16616. doi: 10.1038/s41598-017-16882-5 29192196 PMC5709435

[23] Studach L Wang WH Weber G Tang J Hullinger RL Malbrue R. Polo-like kinase 1 activated by the hepatitis B virus X protein attenuates both the DNA damage checkpoint and DNA repair resulting in partial polyploidy. J Biol Chem (2010) 285:30282–93. doi: 10.1074/jbc.M109.093963 PMC2943266 20624918

[24] Hedrick VE LaLand MN Nakayasu ES Paul LN. Digestion, purification, and enrichment of protein samples for mass spectrometry. Curr Protoc Chem Biol (2015) 7:201–22. doi: 10.1002/9780470559277.ch140272 26331527

[25] Mani SKK Yan B Cui Z Sun J Utturkar S Foca A. Restoration of RNA helicase DDX5 suppresses hepatitis B virus (HBV) biosynthesis and wnt signaling in HBV-related hepatocellular carcinoma. Theranostics (2020) 10:10957–72. doi: 10.7150/thno.49629 PMC7532671 33042264

[26] Sun J Wu G Pastor F Rahman N Wang WH Zhang Z. RNA Helicase DDX5 enables STAT1 mRNA translation and interferon signalling in hepatitis B virus replicating hepatocytes. Gut (2021). doi: 10.1101/2020.09.25.313684 PMC8606016 34021034

[27] Rakotomalala L Studach L Wang WH Gregori G Hullinger RL Andrisani O. hepatitis B virus X protein increases the Cdt1-to-geminin ratio inducing DNA re-replication and polyploidy. J Biol Chem (2008) 283:28729–40. doi: 10.1074/jbc.M802751200 PMC2568909 18693245

[28] Schoggins JW Wilson SJ Panis M Murphy MY Jones CT Bieniasz P. A diverse range of gene products are effectors of the type I interferon antiviral response. Nature (2011) 472:481–5. doi: 10.1038/nature09907 PMC3409588 21478870

[29] Tyanova S Temu T Sinitcyn P Carlson A Hein MY Geiger T. The Perseus computational platform for comprehensive analysis of (prote)omics data. Nat Methods (2016) 13:731–40. doi: 10.1038/nmeth.3901 27348712

[30] Ruepp A Waegele B Lechner M Brauner B Dunger-Kaltenbach I Fobo G. CORUM: the comprehensive resource of mammalian protein complexes–2009. Nucleic Acids Res (2010) 38:D497–501. doi: 10.1093/nar/gkp914 PMC2808912 19884131

[31] Giurgiu M Reinhard J Brauner B Dunger-Kaltenbach I Fobo G Frishman G. CORUM: the comprehensive resource of mammalian protein complexes-2019. Nucleic Acids Res (2019) 47:D559–d563. doi: 10.1093/nar/gky973 30357367 PMC6323970

[32] Will CL Lührmann R. Spliceosome structure and function. Cold Spring Harb Perspect Biol (2011) 3(7):a003707. doi: 10.1101/cshperspect.a003707 21441581 PMC3119917

[33] Jungfleisch J Chowdhury A Alves-Rodrigues I Tharun S Díez J. The Lsm1-7-Pat1 complex promotes viral RNA translation and replication by differential mechanisms. Rna (2015) 21:1469–79. doi: 10.1261/rna.052209.115 PMC4509936 26092942

[34] Díez J Ishikawa M Kaido M Ahlquist P. Identification and characterization of a host protein required for efficient template selection in viral RNA replication. Proc Natl Acad Sci USA (2000) 97:3913–8. doi: 10.1073/pnas.080072997 PMC18116 10759565

[35] Ahlquist P. Parallels among positive-strand RNA viruses, reverse-transcribing viruses and double-stranded RNA viruses. Nat Rev Microbiol (2006) 4:371–82. doi: 10.1038/nrmicro1389 PMC7097367 16582931

[36] Chabrolles H Auclair H Vegna S Lahlali T Pons C Michelet M Couté Y. hepatitis B virus core protein nuclear interactome identifies SRSF10 as a host RNA-binding protein restricting HBV RNA production. PloS Pathog (2020) 16:e1008593. doi: 10.1371/journal.ppat.1008593 33180834 PMC7707522

[37] Kastner B Will CL Stark H Lührmann R. Structural insights into nuclear pre-mRNA splicing in higher eukaryotes. Cold Spring Harb Perspect Biol (2019) 11(11):a032417. doi: 10.1101/cshperspect.a032417 30765414 PMC6824238

[38] Sidarovich A Will CL Anokhina MM Ceballos J Sievers S Agafonov DE. Identification of a small molecule inhibitor that stalls splicing at an early step of spliceosome activation. Elife (2017) 6. doi: 10.7554/eLife.23533 PMC5354520 28300534

[39] Tharun S. Lsm1-7-Pat1 complex: a link between 3' and 5'-ends in mRNA decay? RNA Biol (2009) 6:228–32:e23533. doi: 10.4161/rna.6.3.8282 19279404

[40] Gripon P Rumin S Urban S Le Seyec J Glaise D Cannie I. Infection of a human hepatoma cell line by hepatitis B virus. Proc Natl Acad Sci USA (2002) 99:15655–60. doi: 10.1073/pnas.232137699 PMC137772 12432097

[41] Tremblay MP Armero VE Allaire A Boudreault S Martenon-Brodeur C Durand M. Global profiling of alternative RNA splicing events provides insights into molecular differences between various types of hepatocellular carcinoma. BMC Genomics (2016) 17:683. doi: 10.1186/s12864-016-3029-z 27565572 PMC5002109

[42] Charenton C Graille M. mRNA decapping: finding the right structures. Philos Trans R Soc London Ser B Biol Sci (2018) 373(1762):20180164. doi: 10.1098/rstb.2018.0164 30397101 PMC6232594

[43] Hu X Kim JK Yu C Jun HI Liu J Sankaran B. Quality-control mechanism for telomerase RNA folding in the cell. Cell Rep (2020) 33:108568. doi: 10.1016/j.celrep.2020.108568 33378677

