# Supplementary material for: The cytoplasmic LSm1-7 and nuclear LSm2-8 complexes exert opposite effects on Hepatitis B virus biosynthesis and interferon responses
Source: Front Immunol. 2022 Aug 9;13:970130. doi: 10.3389/fimmu.2022.970130 (PMC9396650; doi:10.3389/fimmu.2022.970130)
Supplement: Supplementary file 1 [file DataSheet_1.docx]

**Supplementary Materials**

**The LSm1-7 and LSm2-8 complexes in Hepatitis B virus biosynthesis**

**and interferon response**

Naimur Rahman^1, 2 +^ Jiazeng Sun^1, 2+^, Zhili Li^1, 2+^, Aryamav Pattnaik^2, 3^, Rodrigo Mohallem^5^

Mangbo Wang^2, 3^ , Majid Kazemian^2, 3, 4^, Uma Aryal^5^ and Ourania Andrisani^1, 2*^

^1^Department of Basic Medical Sciences, ^2^Purdue Center for Cancer Research, ^3^Department of Biochemistry, ^4^Department of Computer Science, and ^5^Department of Comparative Pathobiology Purdue University, West Lafayette, IN 47907, USA

^+^ equal contribution

*corresponding author: [andrisao@purdue.edu](mailto:andrisao@purdue.edu)

Department of Basic Medical Sciences,

Purdue University

201 S. University Street

West Lafayette, IN  47907-2064

Phone: 765-494-8131

**Keywords:** Hepatitis B Virus (HBV), LSm1-7 and LSm2-8 complexes, *N*6-adenosine methylation (m^6^A), Cp028, pgRNA encapsidation.

**Supplementary Data and Figure legends**

**
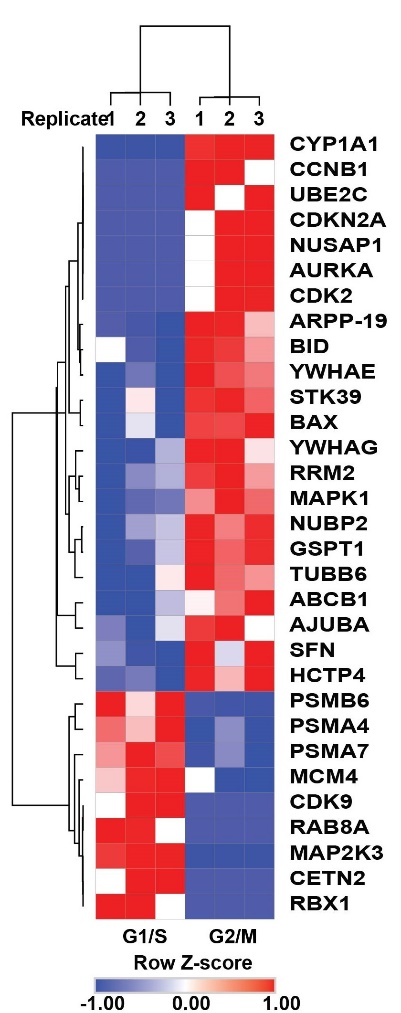
**

**Figure S1: Heatmap representation of proteins associated with G1/S and G2/M cell cycle phase transition during HBV replication.**

HepAD38 cells were synchronized by the double-thymidine block. Heatmap shows G1/S and G2/M cell cycle related proteins (FC≥2, p<0.05) upregulated in G1/S and G2/M phase of cell cycle respectively. Color scale represents Z-scored LFQ values.


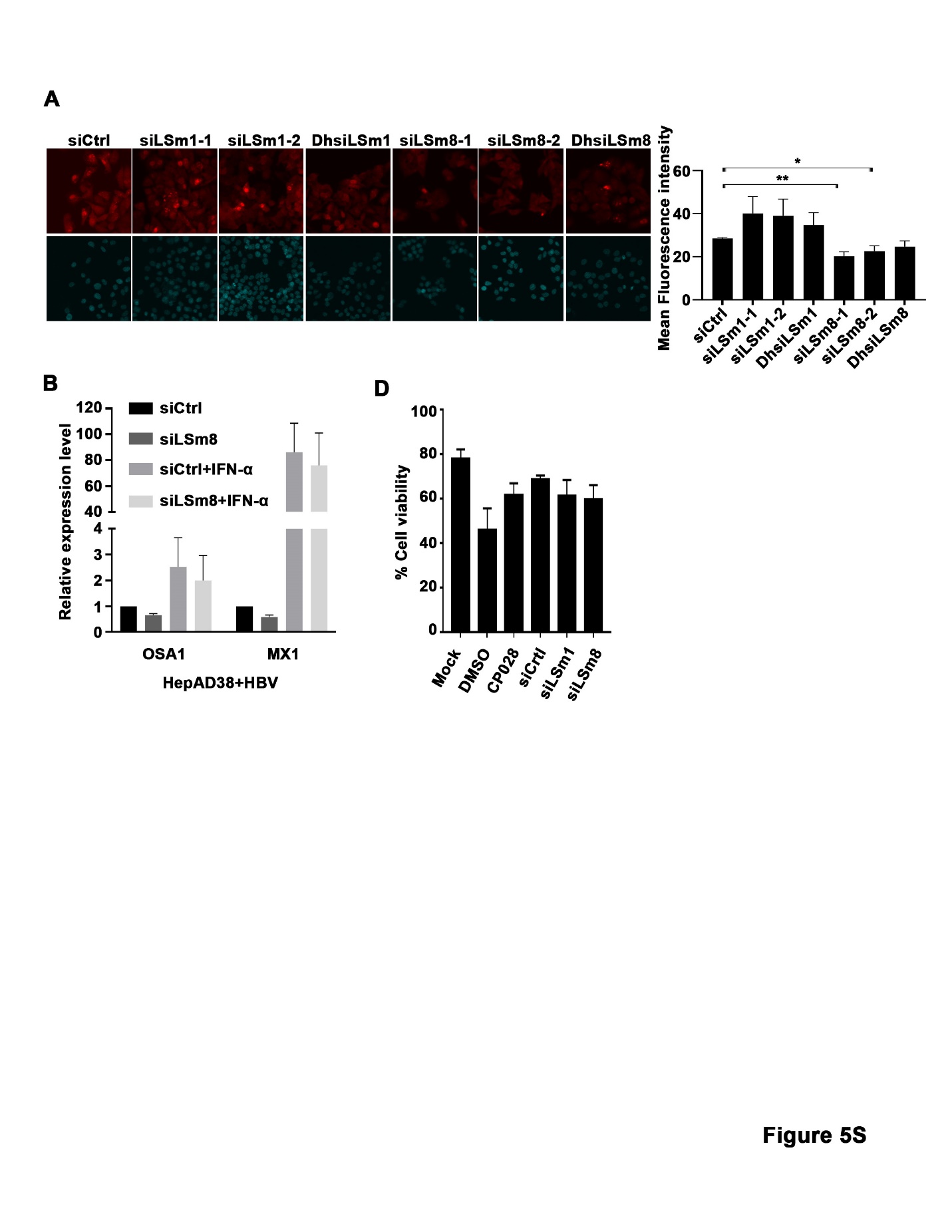


**Figure S2.** **Disruption of LSm complexes does not alter ISG expression in response to IFN-α or** **cell viability.** **A**. HBc immunofluorescence microscopy of unsynchronized HepAD38 cells replicating HBV for 4 days by tetracycline removal, transfected with 50 pM control siRNA (siCtrl), LSm1 siRNAs (siLSm1-1 & siLSm1-2), and LSm8 siRNAs (siLSm8-1 & siLSm8-2) from ThermoFisher, and DhsiLSm1 and DhsiLSm8 from Dharmacon. (Right Panel) Quantification of HBc immunofluorescence by Image J software. **B.** qRT-PCR of ISGs OSA1 and MX1, as a function of LSm8 knockdown and IFN-α treatment in HepAD38 cells. **C.** MTS cell viability assays of HepAD38 cells replicating HBV for 4 days, following siRNA-mediated knockdown of LSm1 and LSm8 or upon treatment with 10µM Cp028.

**
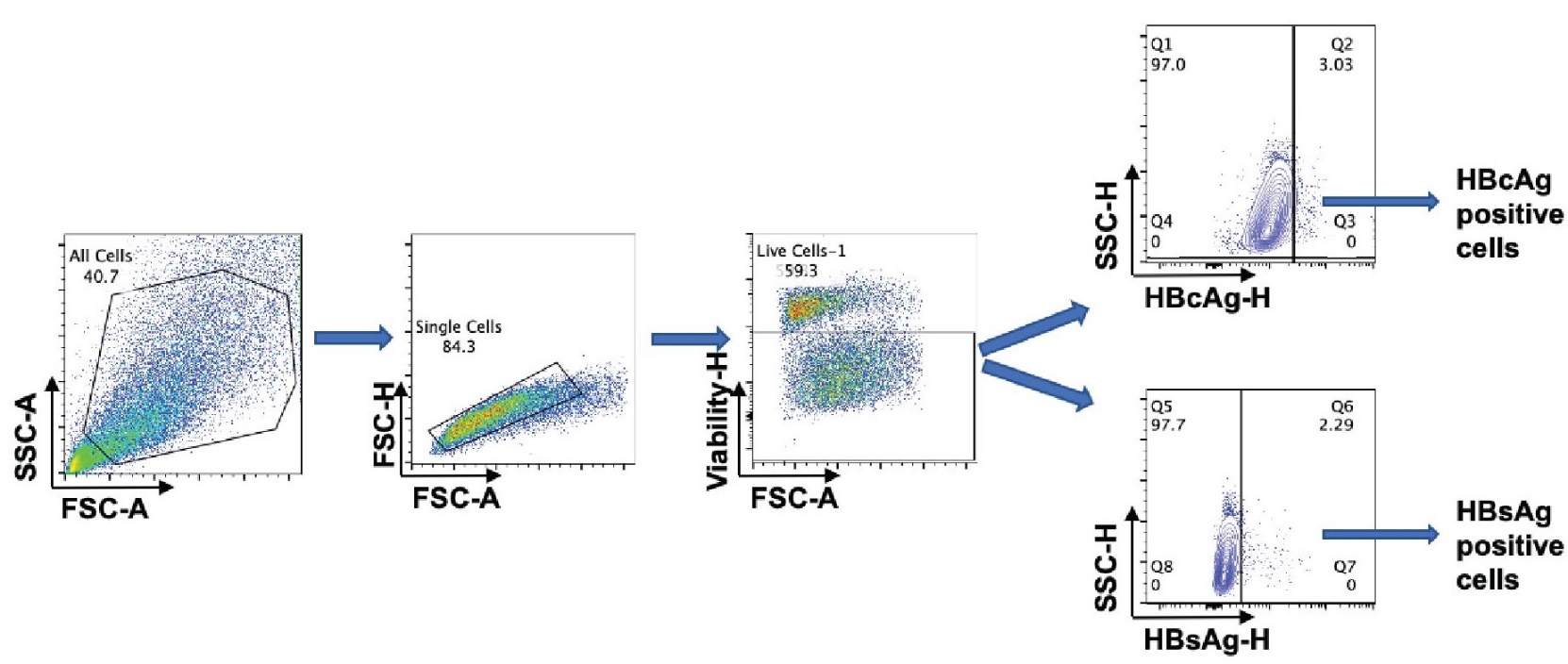
**

**Figure S3 A representative image of the flow cytometry gating strategy demonstrating HBcAg- and HBsAg-positive cells in HBV infected HepG2-NTCP cell population.** Gating strategy for whole cell (SSC-A, FSC-A), Single cell (FSC-H, FSC-A), Live cell (Viability-H, FSC-A), HBcAg-positive cell from live cell population (SSC-H, HBcAg-H) and HBsAg-positive cell from live cell population (SSC-H, HBsAg-H).

**
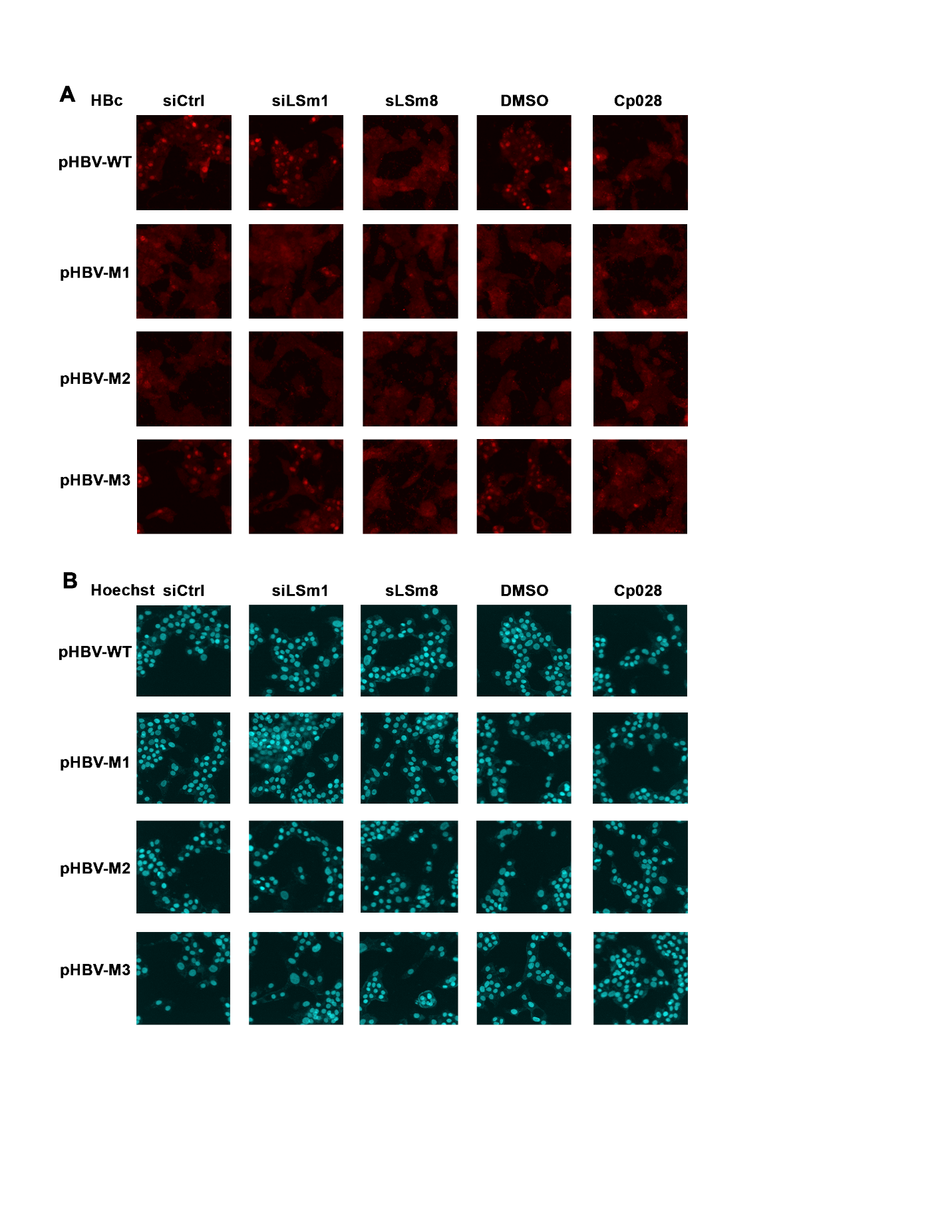
**

**Figure S4.** Immunofluorescence microscopy of HBc in HepG2 cells transfected with indicated 1.3mer HBV plasmids and 50pM of indicated siRNAs or treated with Cp028, as in Figure 5 (Main Text).

**
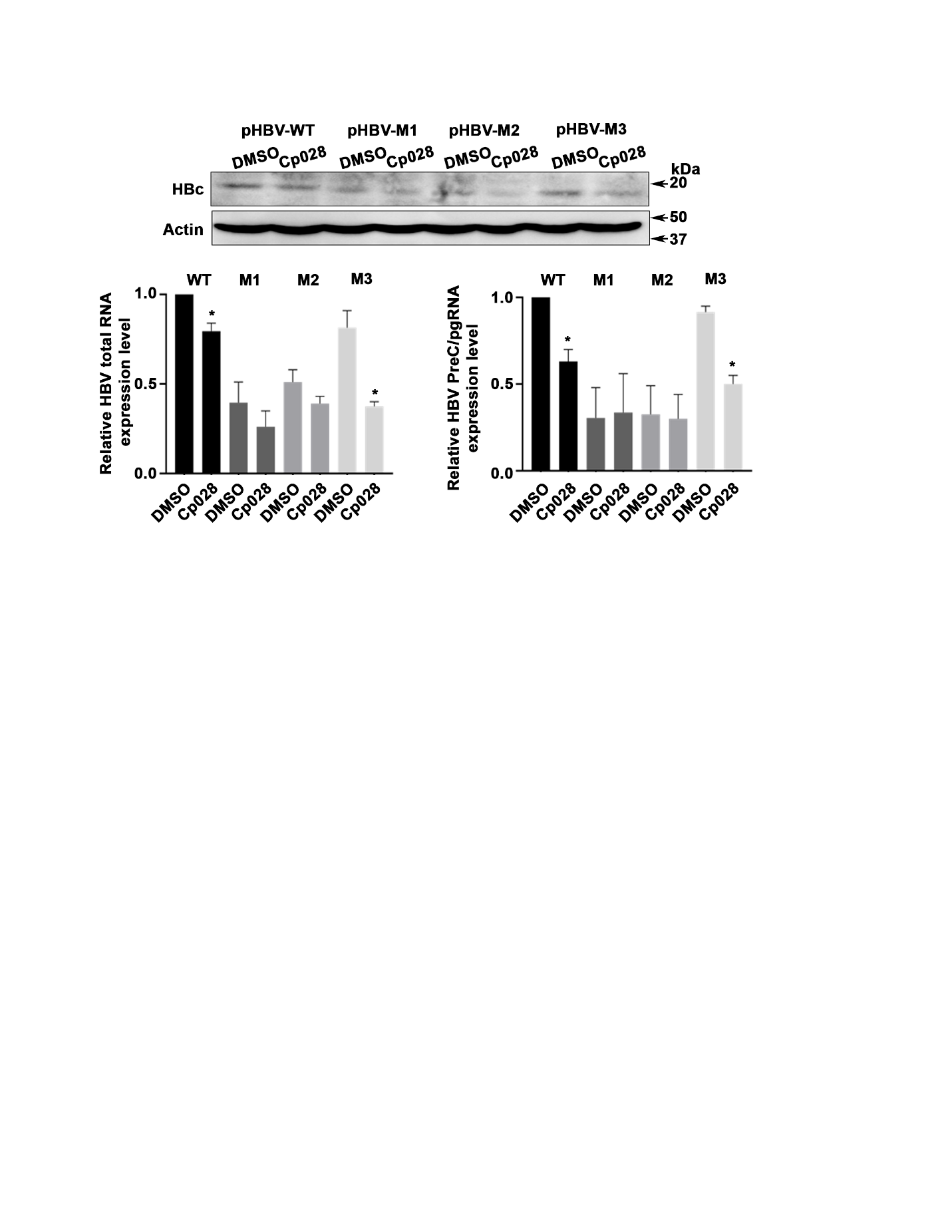
**

**Figure S5**. HepG2 cells were transfected with 1.3mer HBV plasmid in combination with Cp028 (10µM). pHBV-WT, pHBV-M1, pHBV-M2 and pHBV-M3 (1.0µg each per 6-well plate) transfected by lipofectamine3000 in HepG2 cells. HBc immunoblot using lysates of 7day-transfected HepG2 cells. (Lower panels) RNA isolated in parallel from transfected cells used for RT-PCR quantification of total HBV RNA and preC/prRNA. n=3 *p<0.05

**
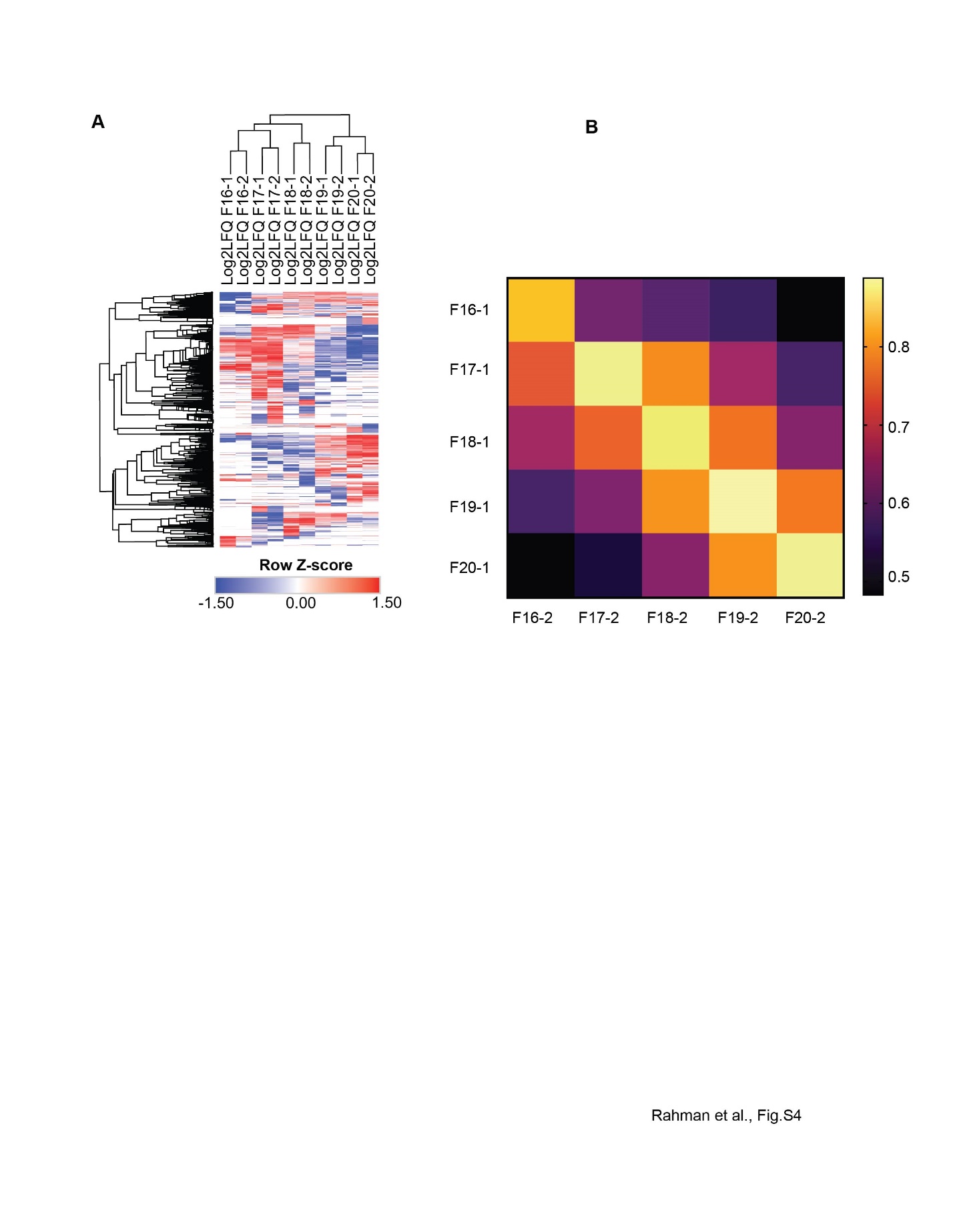
**

**Figure S6 Mass spectrometry of HepaRG nuclear extracts fractionated by size exclusion chromatography. A.** Nuclear extracts of HepaRG cells fractionated by Superdex 30 size exclusion chromatography, fractions 17-20 were analysed by LC MS/MS. Heatmap represents hierarchical clustering of proteins co-eluted in fractions 17-20 in two replicates. Color scale represents Z-scored LFQ values. **B**. Pearson correlation coefficient of biological replicates from each fraction group (n=2). The coefficient in each sample (n=2) ranges from 0.5 to 1.0 where 1.0 indicates a complete correlation.

**Supplementary Methods**

**Cell growth and treatments:** Cell Synchronization in G1/S phase performed by the double thymidine block, using HepAD38 cells grown without tetracycline for 4 days, to enable HBV replication. Cells grown for 16 h in growth medium containing 2.0 mM thymidine (Sigma) followed by incubation for 8 h in medium without thymidine with or without IFN-α (500ng/ml). The second thymidine block was performed for additional 16 h incubation in media containing 2.0 mM thymidine +/- IFN-α (500 ng/ml)(1). For G2/M synchronization, after release from the double thymidine block, cells were grown for 3 h in growth medium +/- IFN-α, followed by 5 h incubation in medium containing nocodazole (400 ng/ml), +/- IFN-α (2). Whole cell extracts (WCE) were prepared from G1/S and G2/M synchronized cultures, and processed for liquid chromatography (LC)-mass spectrometry (MS and MS/MS) exactly as described (3). Three independent WCE preparations similarly prepared and analyzed by LC-MS/MS.

**Nuclear extract preparation:** Nuclear extracts prepared from HepaRG cells, grown with IFN-α (500ng/mL) for 24 h. Cells washed 3X and collected by scraping in ice-cold phosphate-buffered saline (PBS), followed by centrifugation for 5 min at 450 x g. Cell pellets resuspended in hypotonic lysis buffer (10 mM HEPES pH 7.9, 1.5 mM MgCl2, 10 mM KCl, 1 mM dithiothreitol), were incubated on ice for 15 min and collected by centrifugation at 420xg for 5 min; pellets resuspended in hypotonic lysis buffer, homogenized on ice for ten strokes (type B pestle), and centrifuged at 3000 x g for 20 min. The pellet resuspended in extraction buffer, containing 20 mM HEPES pH 7.9, 25% glycerol, 420 mM NaCl, 0.2 mM EDTA, 1 mM DTT and proteinase inhibitors, homogenized on ice with ten strokes (type B pestle), followed by incubation for 30 min at room temperature with gentle shaking. Chromatin pelleted by centrifugation at 20,000x g for 5 min at 4°C, and soluble nuclear protein lysate used immediately for size exclusion chromatography.

**Size Exclusion Chromatography (SEC):** Nuclear lysates were separated on Superdex 200 10/300 GL column (GE Healthcare) using an ÄKTA fast protein liquid chromatography (FPLC) system (Amersham Biosciences). SEC column was equilibrated with buffer A (50 mm Tris-HCl, pH 7.5) overnight. Column calibration performed using protein standards (MWGF1000, Sigma–Aldrich) with a mass range from 29 - 669 kDa. A total of 400 μL nuclear lysate (1.0 mg nuclear protein) was loaded onto the column, and eluted in 1.25 column volumes at a flow rate of 0.2 mL/min. Void volume was measured with blue dextran. SEC separation performed at 6 °C. Fractions analyzed by LC–MS/MS.

**Proteomics Sample Preparation:** Cell pellets were resuspended in 100 mM ammonium bicarbonate and homogenized using a Barocycler 2320 (90 cycles: 35k PSI for 50 seconds and 1 ATM for 10 seconds, at 5°C) (Pressure BioScience Inc.) for 90 seconds at 6500 rpm. After lysis, protein concentration determined by bicinchoninic acid (BCA) assay (Pierce Chemical Co., USA). Fifty (50) µg was precipitated with 4 volumes of cold (-20°C) acetone and incubated overnight at -20°C. Samples centrifuged at 13,500 rpm for 15 minutes at 4°C, supernatant (acetone) discarded, and pellet dried in vacuum. Dried protein pellets were resuspended in 10 µL of 10 mM DTT in 8M urea, and incubated at 37 °C for 1 h. Equal volume of alkylating mixture (195 µL acetonitrile, 1.0 µL triethyl phosphate, 4.0 µL iodoethanol) was added, samples were incubated at 37 °C for 1 h before drying in vacuum. Samples were digested with Lys-C/Trypsin in 25 mM ammonium bicarbonate in a 1:25 enzyme to protein ratio, using a Barocycler (60 cycles: 20k PSI for 50 seconds and 1 ATM 10 seconds, at 50 °C). Digested peptides were desalted using C_18_ Silica MicroSpin Columns (The Nest Group, Inc. USA).

**Liquid Chromatography-Tandem Mass Spectrometry (LC-MS/MS) analysis:** Digested peptides were analyzed on Orbitrap Fusion Lumos Mass Spectrometer (Thermo Fisher Scientific) equipped with a Dionex UltiMate 3000 RSLC nano System (Thermo Fisher Scientific) interfaced via a Nanospray Flex nanoelectrospray source(*46*). Briefly, reverse phase peptide separation was accomplished using a trap column (300 μm ID × 5 mm) packed with 5 μm 100 Å PepMap C18 medium coupled to a 50-cm long × 75 µm inner diameter analytical column packed with 2 µm 100 Å PepMap C18 silica (Thermo Fisher Scientific). Column temperature maintained at 50°C. Mobile phase solvent A was 2% acetonitrile (ACN), 98% water and 0.1% Formic Acid (FA). Mobile phase solvent B was 80% ACN, 20% water and 0.1% FA. Samples in loading buffer (3% ACN, 0.1% FA) loaded to column at a flow rate of 5 µL/min for 5 min, and eluted at a flow rate of 200 nL/min, using a 160-min LC gradient: 6.5 to 27% of solvent B in 110 min, 27-40% of B in next 15 min, 40-100% of B in next 10 min at which point the gradient was held at 100% of B for 10 min before reverting back to 2% of B, and hold at 2% of B for next 15 min for column equilibration. Column further washed and equilibrated using three 30-min LC gradient before injecting next sample. All data acquired in the Orbitrap mass analyzer at resolution of 120,000 for MS1 and 15,000 for

MS/MS at 200 m/z.

**Mass Spectrometry Data Analysis:** LC-MS/MS data were analyzed using MaxQuant software (version 1.6.3.3) against the combined non-redundant human protein sequence database from UniProt, for protein identification and label-free quantitation. The following parameters were used for database searches: precursor mass tolerance of 10 ppm; enzyme specificity of trypsin/Lys-C enzyme allowing up to 2 missed cleavages; oxidation of methionine (M) as a variable modification and iodoethanol (C) as a fixed modification. False discovery rate (FDR) of peptide spectral match (PSM) and protein identification was set to 0.01. Proteins with LFQ # 0 and MS/MS (spectral counts) ≥ 2 considered as identified, and used for statistical analysis and visualization in Perseus platform (*47*). Statistical analysis performed using Analysis of Variance (ANOVA) and two-tailed Student’s t-test. Proteins with a p value ≤ 0.05 and absolute Log2 (LFQ) ≥ 0.50 were considered significantly regulated.

**Hepatitis B Virus Preparation:** HBV was derived from HepAD38 cells, by collecting the growth medium from HepAD38 cells at days 31–90 post-induction of viral replication by tetracycline removal. Medium were cleared through a 0.45- μm filter and precipitated with 10% PEG8000. Precipitates washed and resuspended in medium at 100-fold concentration. HBV DNA quantified by real time PCR.

**HBV Infection:** HepG2-NTCP cells seeded onto collagen-coated 24-well plates, with density of 2×10^5^ cells/well, were grown in regular DMEM/F12 medium supplemented with 10% FBS, 100 U/ml penicillin, and 100 μg/ml streptomycin overnight. Next, they were switched in DMEM, 3% FBS, 1% NEAA (non-essential amino acids), 2% DMSO, 100 U/ml penicillin, and 100 μg/ml streptomycin. After 24 h of 2% DMSO treatment, cells were infected with HBV stock at 100-500 vge/cell. The viral stock was diluted in DMEM supplemented with 0.1mM NEAA, 2% DMSO, and 4% PEG. The inoculum volume was 200 μl /plate; cells were then spinoculated for 1h at 1,000 × g at 37 °C. After 24h, inoculum was removed, cells were washed five times with PBS and maintained in DMEM supplemented with 3% FBS,1% NEAA and 2% DMSO before harvest.

**Flow Cytometry quantification of HBV infected cells:** HBV infected HepG2-NTCP cells were trypsinized and washed with Phosphate bufferred solution (PBS) on day 7 post-infection, and pelleted at 2500 rpm for 5 mins. Subsequently, cells were stained with eBioscience Fixable viability dye eFluor 780, diluted in PBS (1:2000), and incubated on ice for 30 mins, followed by washing with PBS. Cells were incubated with fixation and permeabilization buffer for 30 min at room temperature, and washed with 1x permeabilization buffer. Intracellular staining was carried out by incubating cells with primary HBcAg (1:1000) and HBsAg (1:1000) (invitrogen MA1-7603) antibodies in perm buffer for 40 min at room temperature. Cells were washed twice with permeabilization buffer, incubated with the cocktail of Alexa-546 goat anti-rabbit and Alexa-647 goat anti mouse IgG (Invitrogen A21235) antibodies for 40 min at room temperature. Cells were washed with permeabilization buffer twice, and once with PBS and permeabilization (1:1), fixed with 2% formaldehyde for 20 mins, washed with permeabilization buffer and analyzed by flow cytometry in an Attune NxT Flow Cytometer (Thermo Fisher). Data analysis performed using FlowJo software 10.8.1. Representative images of flow cytometry gating strategy shown in Supplementary Fig S6.

**Supporting Table S1: List of Plasmids and siRNAs**

| **Plasmids, siRNAs** | **Source** |
| --- | --- |
| pHBV-WT | A gift from Dr. Aleen Siddiqui’s lab |
| pHBV-M1 | A gift from Dr. Aleen Siddiqui’s lab |
| pHBV-M2 | A gift from Dr. Aleen Siddiqui’s lab |
| pHBV-M3 | A gift from Dr. Aleen Siddiqui’s lab |
| siCtrl | ThermoFisher Scientific (#4390843) |
| siLSm1 | ThermoFisher Scientific (AM16708, assay id 134362) |
| siLSm8 | ThermoFisher Scientific (AM16708, assay id 134872) |
| DhsiLSm1 | Dharmacon (L-005124-00-0005) |
| DhsiLSm8 | Dharmacon (L-017030-01-0005) |

**Supporting Table S2: Antibodies**

| **Antibody** | **Dilution** | **Application** | **Source** |
| --- | --- | --- | --- |
| Rabbit α-HBV Core | 1:5000 in 2% BSA in TBST/1:1000 in 2% BSA in PBS/1:1000 in Perm buffer | Western Blot/Immunofluorescence/Flow cytometry | Dr. Adam Zlotnick lab |
| Mouse LSm8 | 1:5000 in 2% BSA in TBST/5 μg | Western Blot/ Immunoprecipitation | Santa Cruz (#sc-390542) |
| Rabbit LSm3 | 1:5000 in 2% BSA in TBST | Western Blot | NOVUS (#NBP3-04399) |
| Mouse α-Human Actin | 1:1000 in 2% BSA in TBST | Western Blot | Sigma (#A5441) |
| Rabbit α-Human STAT1 | 1:1000 in 2% BSA in TBST | Western Blot | Cell Signaling Technologies (#14994S) |
| Rabbit α-Human p-STAT1 | 1:1000 in 2% BSA in TBST | Western Blot | Millipore Sigma (#07-307) |
| Rabbit α-Human IRF9 | 1:1000 in 2% BSA in TBST | Western Blot | Cell Signaling Technologies (#76684S) |
| Horse α-Mouse secondary | 1:2000 in 2% BSA in TBST | Western Blot | Vector Laboratories (#PI-2000) |
| Goat α-Rabbit secondary | 1:2000 in 2% BSA in TBST | Western Blot | Vector Laboratories (#PI-1000) |
| Mouse HBsAg | 1:1000 | Flow Cytometry | ThermoFisher Scientific (#MA1-7603) |
| Rabbit IgG | 5 μg | Immunoprecipitation | Millipore Sigma (#17-700) |
| Mouse IgG | 5 μg | Immunoprecipitation | Millipore Sigma (#17-700) |
| Goat 546-Rabbit secondary | 1:1000 in 2% BSA in PBS | Immunofluorescence | Invitrogen (#02189179) |
| M6A antibody | 3µg/RNA sample | MeRIP | Synaptic Systems  (#202003) |
| Rabbit IgG | 3µg/RNA sample | MeRIP | Cell Signaling Technology (#2729S) |

**Supporting Table S3: Primer and RNA oligonucleotide sequences**

| **Primer** | **5’ – Sequence – 3’** |
| --- | --- |
| GAPDH-F | CCCTTCATTGACCTCAACTACA |
| GAPDH-R | ATGACAAGCTTCCCGTTCTC |
| Actin-F | GGCATGGGTCAGAAGGATT |
| Actin-R | GGGGTGTTGAAGGTCTCAAA |
| UBc-F | CCTGGAGGAGAAGAGGAAAGAGA |
| UBc-R | TTGAGGACCTCTGTGTATTTGTCA |
| HBV total RNA-F | TCACCAGCACCATGCAAC |
| HBV total RNA-R | AAGCCACCCAAGGCACAG |
| HBV pgRNA-F | GAGTGTGGATTCGCACTCC |
| HBV pgRNA-R | GAGGCGAGGGAGTTCTTCT |
| LSm1-F | GGTCAGAGGAGAAAATGTGGTCC |
| LSm1-R | CTTGGTCTGCTGTTCCACCCTT |
| LSm3-F | CTCAGCCTAGATGAGCGAATTTAT |
| LSm3-R | AGTAGTCACAGTTTCTTCCACATC |
| LSm8-F | AGTAGTCACAGTTTCTTCCACATC |
| LSm8-R | CCCAAATCAAGCGCAGAATCTGT |
| MX1-F | GGCTGTTTACCAGACTCCGACA |
| MX1-R | CACAAAGCCTGGCAGCTCTCTA |
| OSA-F | AAGCCACCAACTCCAGCATCCA |
| OSA-R | CGCTTCTGGAATGTGGAGTCAC |

**Supporting Table S4: Reagents, Chemical inhibitors, and Kits**

| **Reagents, Chemical inhibitors, Kits** | **Source** |
| --- | --- |
| Cp028 | Glixx (#347397-83-5) |
| IFN-α | Millipore Sigma (#SRP4596) |
| PEG8000 | Sigma (#P5413) |
| Hoerchst 33342 | ThermoFisher Scientific (#62249) |
| MTS | Promega (#G3580) |
| PCR Mycoplasma Detection Kit | Abm (#G238) |
| Cell Lysis Buffer (10X) | Cell Signaling Technology (#9803) |
| LightCycler® 480 SYBR Green I Master | Roche (#04887352001) |
| iScript™ cDNA Synthesis Kit | Biorad (#1708891) |
| Nitrocellulose Membrane, Roll, 0.2 µm | Biorad (#1620112) |
| LightCycler® 480 Sealing Foil | Roche (#04729757001) |
| LightCycler® 8-Tube Strips (white) | Roche (#06612601001) |
| DMSO | Sigma (#D8418-50ML) |
| Tween™ 20 | ThermoFisher Scientific (#BP337-500) |
| Tetracycline hydrochloride | Sigma (#T7660-5G) |
| Bovine Serum Albumin | Sigma (#A9647-100G) |
| Pierce™ ECL Western Blotting Substrate | ThermoFisher Scientific (#32106) |
| Pierce™ BCA Protein Assay Kit | ThermoFisher Scientific (#23227) |
| Lipofectamine™ 3000 Transfection Reagent | ThermoFisher Scientific (#L3000015) |
| Lipofectamine™ RNAiMAX Transfection Reagent | ThermoFisher Scientific (#13778150) |
| Restore™ PLUS Western Blot Stripping Buffer | ThermoFisher Scientific (#46430) |
| RNeasy Mini Kit | Qiagen (#74104) |
| Magna RIP™ RNA-Binding Protein Immunoprecipitation Kit | Millipore Sigma (#17-700) |

References

1. Rakotomalala L, Studach L, Wang WH, Gregori G, Hullinger RL, Andrisani O. Hepatitis B virus X protein increases the Cdt1-to-geminin ratio inducing DNA re-replication and polyploidy. J Biol Chem 2008;283:28729-28740.

2. Studach L, Wang WH, Weber G, Tang J, Hullinger RL, Malbrue R, Liu X, et al. Polo-like kinase 1 activated by the hepatitis B virus X protein attenuates both the DNA damage checkpoint and DNA repair resulting in partial polyploidy. J Biol Chem 2010;285:30282-30293.

3. Hedrick VE, LaLand MN, Nakayasu ES, Paul LN. Digestion, Purification, and Enrichment of Protein Samples for Mass Spectrometry. Curr Protoc Chem Biol 2015;7:201-222.
